# Effect of Graphene on the Mechanical Properties of Recycled High-Density and High-Molecular-Weight Polyethylene Blends

**DOI:** 10.3390/ma17194733

**Published:** 2024-09-26

**Authors:** Hniya Kharmoudi, Alae Lamtai, Said Elkoun, Mathieu Robert, Carl Diez

**Affiliations:** 1 Center for Innovation in Technological Eco-Design (CITE), University of Sherbrooke, Sherbrooke, QC J1K 2R1, Canada; hniya.kharmoudi@usherbrooke.ca (H.K.); alae.lamtai@usherbrooke.ca (A.L.); mathieu.robert2@usherbrooke.ca (M.R.); 2 Research Center for High Performance Polymer and Composite Systems, CREPEC, Montréal, QC H3A 0C3, Canada; 3 Soleno Inc. Maitrise de l’Eau Pluviale, Saint-Jean-sur-Richelieu, QC J2X 4B6, Canada; carl.diez@soleno.com

**Keywords:** recycled high-density polyethylene, recycled high molecular weight, graphene, mechanical, thermal properties, stress crack resistance

## Abstract

This study uses an extrusion process to formulate blends based on recycled high-density and high-molecular-weight polyethylene (recHDPE, recHMWPE) for the manufacture of rainwater drainage pipes. The main objective of this project is to investigate the effects of incorporating graphene on the mechanical, thermal, and stress-cracking resistance properties of the recycled HDPE and HMWPE blends. Also, it aims to demonstrate that the addition of graphene may enable the use of different recycled polymers without compromising their properties. The effects of adding two amounts of graphene (0.5 and 1%) to recycled blends on the tensile and flexion properties, stress crack resistance (SCR) (using a notched crack ligament stress (NCLS) test), thermal behavior (using a differential scanning calorimeter (DSC) and a rheological plastometer) were investigated. The experimental results showed a significative enhancement when adding graphene in the SCR, some tensile properties (elongation at break and tensile strength), and flexural modulus. However, physical characterization showed that the samples containing 0.5% graphene exhibited lower crystallinity compared to the reference and, for the blend with 1% graphene, the fluidity also decreased for the blend filled with the graphene compared to the reference blend without any filler.

## 1. Introduction

Plastic products are omnipresent in our daily lives thanks to their advantages such as resilience, easy processing, low cost, durability, and impact resistance. The problem is that these multiple benefits can threaten the environment, since plastic waste is not reused. Worldwide, plastic production has grown over the last decade; it reached 400.3 million metric tons (Mt) in 2022 [1]. Unfortunately, most consumer plastics are intended to be single-use, with limited recyclability, leading to increased worldwide production and plastic waste consumption [2,3]. The treatment of plastic waste has become a real issue, especially after the COVID-19 crisis [4]. This is why the development of efficient recycling methods for plastic waste is necessary to promote the circular economy. Recycling is the result of the different stages of collection, sorting, washing, and processing of polymers [5].

Ensuring good properties for recycled products is not always guaranteed, since the properties of recycled plastics are generally not as good as those of virgin resins [6], which limits the reuse of these plastic wastes in various industrial applications [7,8]. Also, various problems are encountered during the recycling process due to polymer degradation and the incompatibility of different polymer types. Several additives are used to remedy these problems and improve the plastic’s properties, such as antioxidants (AOs), which are chemical compounds that protect polymers against the thermal oxidation process [9]. They can interfere with the oxidative cycles to delay the oxidative degradation of polymer blends [8,9]. Fillers and modifiers are compounds added to the processing stage to improve the mechanical properties of the polymer blends. Fillers enhance the modulus and tensile strength but worsen the processability and elongation at break. In comparison, modifiers improve the elongation at break and impact strength [5]. Heat stabilizers, widely used in PVC blends, are added to prevent the thermal degradation of plastics [9]. Plasticizers, as organic substances of low volatility, are used to improve the flexibility and processability of plastic materials [9]. The use of compatibilizers is essential to exhibit interfacial activities in heterogeneous recycled polymer blends, and carbon black (CB) is considered as a reinforcing filler used to boost dimensional stability, as well as an antioxidant to extend service life [8,10]. Additives are crucial compounds of plastic materials, enabling the performance and modification of the polymer’s properties and long-term use. The main objective of adding these components in the recycling stage is to prevent degradation phenomena during the recycling process and to improve the properties of the extruded materials generated in the recycling process [11].

To improve the mechanical properties of polymer blends based on recycled post-consumer high-density polyethylene (HDPE) and high-molecular-weight polyethylene (HMWPE), carbonaceous nanofillers such as carbon nanofibers, carbon nanotubes, and graphene nanoplatelets are widely used [5,12,13,14,15]. Since discovering graphene in 2004 [16], and thanks to its high mechanical performance, graphene has become a promising filler for the development of polymer composites with good mechanical properties [17].

Graphene is a two-dimensional (2D) allotrope of carbon consisting of a single layer of carbon atoms with a honeycomb lattice [18,19] bonded via weak Van der Waals forces [20] that has been found to have exceptional mechanical [19,21], electrical [19,21], and thermal properties [22,23]. Due to its exceptional two-dimensional structure, graphene has excellent mechanical properties, an ultimate tensile strength of 130 GPa, and a Young’s modulus of 1TPa [24], with a high thermal conductivity of 5000 w/(m.k), displaying high mobility of charge carriers [17].

Incorporating graphene as a micro-filler in a polymer matrix improves the mechanical properties of polymer composites [25]. The addition of graphene to the polymer increased the Young’s modulus and tensile strength of the polymer blend [26,27]. Moreover, Wang et al. compared the effect of adding graphite and carbon black to high-density polyethylene (HDPE) [28], and the results showed that HDPE/graphite improved tensile and impact strength better than HDPE/carbon black. Other papers have highlighted the effect of incorporating exfoliated graphite nanoplatelets into polypropylene (PP); this additive enhanced the dimensional stability and rheological behavior of the material.

Yassmin et al. studied the effect of adding graphene to an epoxy matrix; they concluded that the addition of this nanofiller increased the elastic modulus [29]. Another study, conducted by Gupta et al. [30], showed that the incorporation of graphene in vinyl ester nanocomposites significantly improve the storage modulus, loss modulus, and glass transition temperature of this material.

This study is conducted on an innovative industrial decontamination line. It investigates the mechanical and physical properties of a recycled HDPE and HMWPE blend reinforced with graphene. The composites were processed using an industrial twin-screw extruder. The effects of the processing parameters, such as filler concentration, were experimentally analyzed through thermal and rheological characterization, tensile and flexural tests to assess the mechanical behaviour, and notched crack ligament stress (NCLS) to evaluate the stress crack resistance of the PE blend (SCR).

## 2. Materials and Methods

### 2.1. Materials

#### 2.1.1. Polyethylene

Recycled polyethylene blends were prepared via a twin-screw extruder using recycled HDPE (recHDPE) and recycled HMWPE (recHMWPE). The reference blend is based on 35% recHDPE and 65% recHMWPE.

The RecHDPE used in this study is a post-consumer recHDPE with a melt flow index of 0.5 g/10 min (190 °C/2.16 kg load) and a density of 0.951 g$/{\mathrm{cm}}^{3}$. The RecHMWPE is also a post-consumer plastic with a melt flow of 0.1 g/10 min (190 °C/2.16 kg load) and a density of 0.948 (g/${\mathrm{cm}}^{3}$), Soleno inc., Saint-Jean sur Richelieu, QC, Canada, provided all these materials.

#### 2.1.2. Graphene

The filler used in this study consists of graphene particles and is a multifunctional carbon additive formulated for across-the-board performance enhancements in thermoplastics and rubbers. The graphene was supplied in the form of a masterbatch based on medium-density PE (MDPE) filled with graphene Black 0X. Graphene pellets 0X are two-dimensional (2D) with 6 to 10 layers, a primary particle size of 0.5–1 µm, and a density of 0.2–0.3 g/${\mathrm{cm}}^{3}$.

Graphene was added to the reference blend in two different amounts (0.5 and 1%). Its composition and technical properties are presented in Table 1, Table 2 and Table 3 below:

### 2.2. Methods

#### 2.2.1. Preparation of Blends

The preparation of blends consists of extruding the pellets using a co-rotating twin-screw extruder at 1200 rpm, with a temperature profile between 190 and 210 °C. Initially, a premix of recHDPE with 5 wt% of the graphene masterbatch was prepared using an electric mixer. Subsequently, the prepared premix was added to the reference blend based on 35% recHDPE and 65% recHMWPE via a feeder. Two amounts of graphene masterbatch were tested (0.5 and 1 wt%) in the extrusion process. The main objective is to test the effect of adding (0.5 and 1 wt%) graphene on the thermomechanical and stress crack resistance properties of recHDPE and recHMWPE blends. The compositions of the extruded blends are illustrated in the Table 4 below:

#### 2.2.2. Samples Preparation

Samples for mechanical characterization (Tensile, Flexion, NCLS, IZOD) were prepared using a press machine at 180 °C for approximately 20 min. The molded plates were cooled to 80 °C; once the plates were ready, they were placed in a conditioning room (21 ± 2 °C) for 24 h before starting the characterization.

### 2.3. Physical Characterization

#### 2.3.1. Mechanical Properties

**Figure 1:**
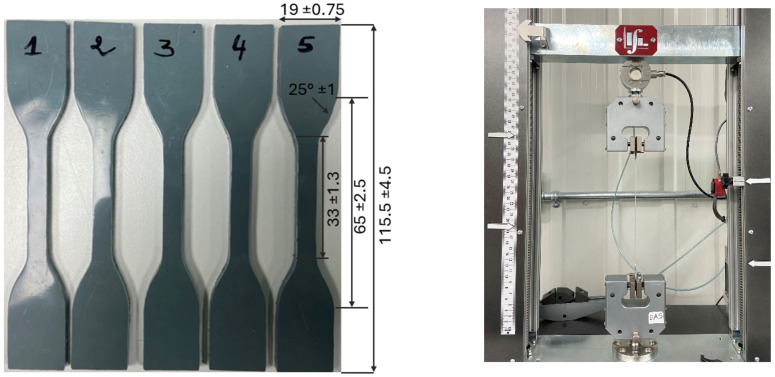
Dimensions (mm) of tensile specimens.

The mechanical behavior was assessed using tensile and flexural tests. Tensile tests were performed based on the ASTM D638-14 [33] on five dog-bone-shaped specimens (specimen type IV: Figure 1) cut from the 3.2 mm thick molded plate. Tensile tests were performed on a Lab Integration machine with a crosshead speed of 50 mm/min at room temperature 23 °C; five specimens were tested for each test. Tensile strength, elongation at break, and Young’s modulus were determined from stress–strain curves. Bending tests were carried out on the same machine as the tensile test in three-point bending mode with a crosshead speed of 10 mm/min according to ASTM D790 [34] on five rectangular specimens to determine the flexural modulus.

Resistance to crack propagation was also assessed using the notched constant ligament stress (NCLS) test according to the F2136-18 standard [35]. This test method is used to evaluate the crack resistance under constant ligament stress for high-density polyethylene resins or corrugated pipelines. The NCLS test subjects a dumbbell-shaped notched test specimen to constant ligament stress in the presence of a surface-active agent (lgepal) at an elevated temperature [35]. The purpose of this test is to measure the failure time associated with each dumbbell-shaped notched test specimen.

The NCLS test consists of notching five test specimens, then determining the load to be placed on each specimen and loading the weight tubes with a shot. Before attaching the shot tube to the lever arm, we must attach the specimens to the loading frame and place them into the bath conditioned at a temperature of 50 ± 1 °C for 30 min. Then, the weight is connected by a tube to the lever arm for each specimen and the specimen timer is begun immediately. The time to failure for each specimen was recorded.

**Figure 2:**
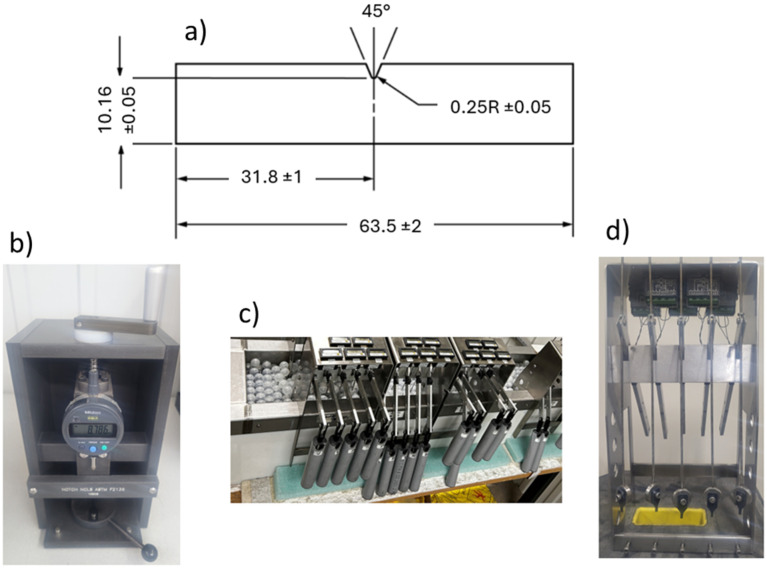
Steps of the NCLS test. Specimen (**a**) dimension [36], (**b**) notching, (**c**) loading in the test basin, and (**d**) failure.

The impact resistance of blends was evaluated using the IZOD test according to the ASTM D256-10 standard [36]; its main objective is to measure the energy absorbed by a material when a notched specimen is subjected to a sudden impact load. The IZOD test consists of notching and testing the 8 notched specimens with a V-shaped notch using a pendulum. Figure 2 illustrates (a) sample preparation, specimens (b) loading, (c) failure and (d) dimensions of the specimen. During the test, the energy absorbed by the specimen indicates the impact resistance and toughness of the material.

#### 2.3.2. Thermal Properties

Thermal analysis was realized using a differential scanning calorimeter (DSC) according to ASTM-D3895-14 [37] using a TA instruments DSC. Samples of 5–10 mg were encapsulated in an aluminium pan and heated from the ambient temperature to 80 °C in an inert gaseous environment (nitrogen) at a constant rate of 10 °C/min. Then, the samples were isothermally kept at 80 °C for 1 min before heating again up to 200 °C.

#### 2.3.3. Rheological Properties

The melt flow index measures the fluidity of a polymer; this test method consists of determining the rate of extrusion of molten thermoplastic resins using an extrusion plastometer. The preheated resin is extruded through a die with a specific length and orifice diameter under a specific load, temperature, and piston position in the barrel. The fluidity was evaluated using a Lab Integration plastometer [32].

## 3. Results and Discussion

The composite blends were manufactured using 35% recHDPE and 65% recHMWPE as a matrix and graphene as an additive. Their mechanical, thermal, and stress cracking propagation properties were studied.

### 3.1. Melt Flow Index (MFI)

The fluidity of the extruded blends was realized to evaluate the effect of graphene on the MFI. After adding the graphene, the MFI decreases from 0.177 for the reference blend without any filler to 0.148 or 0.149 g/10 min for the blends with 0.5% graphene or 1% graphene, respectively. This decrease can be explained by the increase in the viscosity of the blends due to reduced chain mobility and enhanced molecular entanglement when adding the graphene. The graphene acts as a reinforcement filler in the polymer, providing a higher viscosity and therefore a lower MFI. It does not significantly affect the viscosity.

### 3.2. Tensile and Flexion Test

Additives play an important role in the processability and applications of plastic materials. Incorporating additives can improve the properties of the plastic materials and make them suitable for different applications [11].

**Figure 3:**
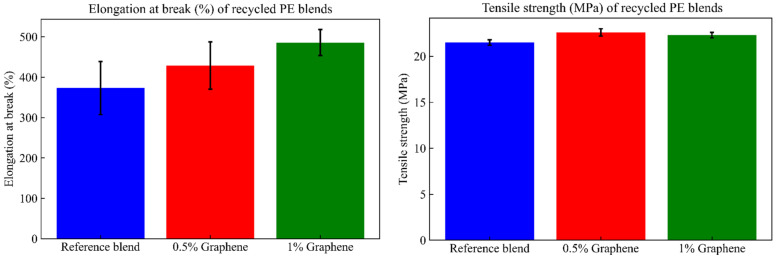

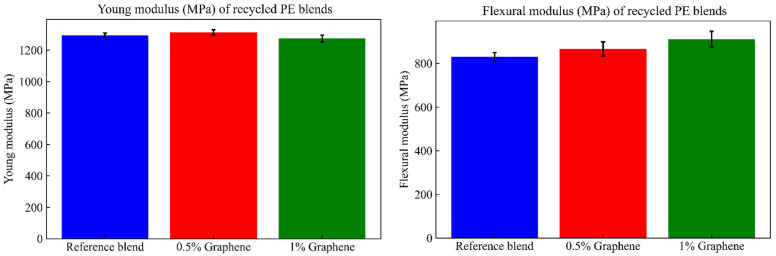
Tensile and flexural properties of PE blends with and without graphene.

It is important to evaluate the mechanical behavior by determining the strength and rigidity of the blends. To do this, tensile and flexion were realized. Figure 3 shows the tensile and flexural properties for the reference blend without graphene and the blends with 0.5% graphene and 1% graphene.

It can be observed that the tensile properties of the reference blend without graphene are lower than those of the blends with graphene. Adding 0.5% or 1% graphene to the reference blend significantly improves the elongation at break by 15% or 30%. Similar results were obtained for the tensile strength and Young’s modulus, which had increases of 5% and 4% for blends with 0.5 and 1% of graphene. The Young’s modulus increases slightly by 2% for blends with 0.5% graphene and decreases by 2% for blends with 1% graphene. These results show that the ductility of the recycled PE blends increases significantly when increasing the amount of graphene. Comparable effects were observed for the flexural properties, with increases of 4% and 10% in the flexural modulus for blends with 0.5% and 1% of graphene, respectively. The same results were reported by Diallo A.K. et al. in their study [6], where there was an improvement of 7% and 12% in the flexural modulus and 6% and 7% in the tensile strength, respectively, when incorporating 1% few-layer graphene (FLG) in a 1:4 ratio of prime/recHDPE and 1:1 ratio of prime/recHDPE [6]. The authors explained that this result is consistent with the SEM images, showing a more homogeneous and refined blend morphology in the presence of FLG. Also, smaller droplet domains help increase the mechanical performance [6].

Adding graphene to recycled HDPE/HMWPE blends increases the mechanical properties because microparticles transfer more tension and improve the connectivity of the composite components [38]. Thanks to the good mechanical properties of graphene and its very high surface area, even small amounts of graphene mixed with polymers can increase the mechanical properties of the blends [38]. The method of adding and choosing the required amount of graphene has a direct impact on the final properties of the blends, exceeding a specified quantity of graphene causes plates to form over each other and the reduction of adhesion between the components of the blends. In general, using low amounts of graphene is an advantage because it avoids agglomeration, which can decrease the mechanical properties. For example, adding up to 0.8 wt% graphene to a wood-fiber-recycled polypropylene composite increases its mechanical strength. On the other hand, the properties are reduced by the addition of 5 wt% graphene [39,40].

### 3.3. Stress Crack Resistance

**Figure 4:**
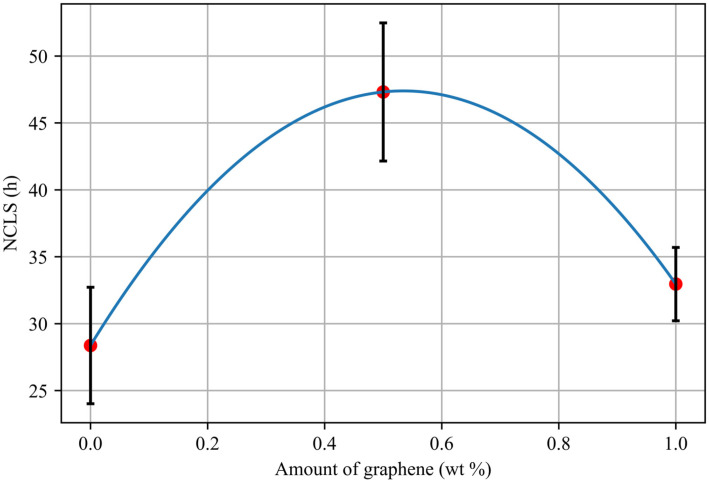
NCLS tests of PE blends as a function of graphene amount.

Evaluating the stress crack resistance of the blends is critical, since toughness is a crucial mechanical property extremely relevant to pipeline applications. To meet the standards required by the BNQ (Bureau de la normalisation du Québec), the minimum break time is 24 h. The result from NCLS tests of the blends without and with graphene are illustrated in the graph below (Figure 4):

Crack resistance is an important factor affecting the long-term sustainability of polymers. The stress crack resistance is affected by the incorporation of graphene. It was observed that adding graphene increases the stress crack resistance by 73% and 20%, respectively, for 0.5 and 1% of graphene. The significant increase related to adding 0.5% of graphene can be explained by the reinforcing effect of graphene, which can potentially slow down crack propagation.

Using graphene as an additive enhances resistance to crack propagation because graphene hinders crack propagation, facilitating stress redistribution and delaying crack propagation. However, adding 1% graphene to the reference blend did not significantly improve the stress crack resistance. With the increase in graphene amount, the microcrack zones become closer to each other, resulting in the coalescence of microcracks, which facilitates the propagation of a major crack [17]. Several studies have proven that the addition of graphene simultaneously enhances stiffness, toughness, and ductility [41].

### 3.4. Impact Strength Resistance

**Figure 5:**
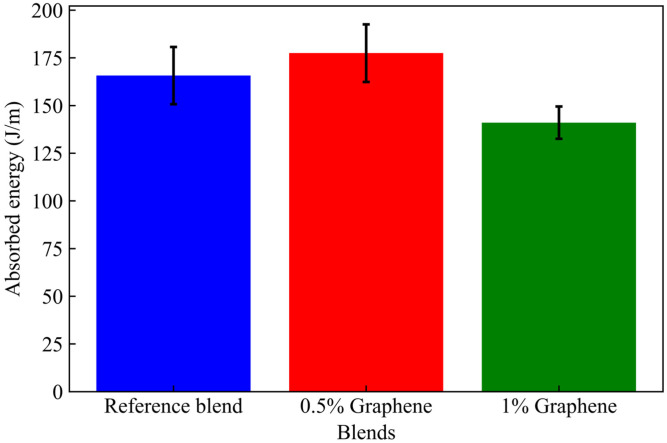
Impact strength resistance of recycled PE blends.

The effect of adding graphene to the reference blend on the impact strength resistance is illustrated in the graphic bellow (Figure 5):

The impact strength resistance of the reference blend without graphene was 165.69 J/m. Adding 0.5% graphene to the reference blend slightly increased the impact strength resistance by 7%. In contrast, the addition of 1% graphene decreases the impact strength resistance by 15%. The observed decrease is due to the agglomeration of the filler. The addition of 1% graphene into reference blend increased the brittleness along with the proportion to rigidity, as shown in the flexural modulus results (Figure 3). Adding graphene to the reference blend may lead to more voids, which decreases the impact strength resistance [41]. The obtained results align with other studies [38,42].

### 3.5. Thermal Behavior

During the processing of polyolefin blends, thermo-oxidative degradation occurs within the polymeric chains, resulting in a finished product of inferior quality [6]. Some of the mechanisms activated during the thermo-oxidative degradation are cross-linking, chain scission, elimination of substituents, and formation of double bonds [43].

The thermal behavior of the blends and the effect of adding graphene were investigated using a DSC. The melt peak temperature and enthalpy were determined directly from the DSC curves, while the degree of crystallinity of blends was calculated from Equation (1). All the data are presented in Table 5.

$\Delta H_{f}^{0}$ presents the enthalpy of fusion of a 100% crystalline polyethylene estimated in the literature at 293 J/g [44].

(1) $$\chi_{c}=\frac{\Delta H_{f}}{\Delta H_{f}^{0}}$$

where $\Delta H_{f}$ is the melting enthalpy of the sample measured during the test (in J/g) and $\Delta H_{f}^{0}$ represents the melting enthalpy of a 100% crystalline polyethylene estimated in the literature at 293 J/g [44].

The results show that the melting peaks of blends increased slightly (by 1 to 2 °C). As a result, the melting enthalpy increased by 3.2 to 9.4 °C, resulting in an enhancement of the degree of crystallinity by 2% and 7% for blends with 0.5% and 1% of graphene, respectively. Since the degree of crystallinity of these polymer blends is less than 50%, the amorphous regions are larger than the crystalline regions, which provides them more flexibility, allowing them to stretch further before breaking and resulting in increased elongation at break. Adding graphene reduces the degree of crystallinity and, contrarily, increases the elongation at break, as demonstrated by the tensile test. The degree of crystallinity affects the physical properties as well as the mechanical properties of semicrystalline polymers.

## 4. Conclusions

In this study, we investigated the effect of adding graphene to a reference blend based on recHDPE and recHMWPE. The purpose of this study was to assess the effect of adding graphene on the thermomechanical behavior and the stress crack resistance of the reference blend. The experimental tests showed that adding graphene improves tensile strength, elongation at break, and flexural modulus. Furthermore, stress crack and impact strength resistance are enhanced when using low amounts of graphene (0.5%). Adding graphene to recycled polyethylene blends could be an interesting alternative to ensure good mechanical properties and resistance to crack propagation that is comparable to virgin PE blends.

Moreover, rheological and microstructural experiments are being performed to evaluate the effect of adding graphene on the interfacial adhesion between the different phases of the blend in the presence of graphene.

## Figures and Tables

**Table 1:** Graphene masterbatch composition.

Composition		Loading (wt%)
Carrier	MDPE	70
Filler	Graphene Black	30

**Table 2:** Chemical composition of graphene 0X.

Element	Value (wt%)
Carbon	>97
Oxygen	<1

**Table 3:** Technical properties of graphene masterbatch.

Property	Value (SI)	Test Method
Density	1.12 g/${\mathrm{cm}}^{3}$	ASTM D792 [31]
Fluidity	8 g/10 min	ASTM D1238 [32]

**Table 4:** Blends composition.

Blend Number	recHDPE (wt%)	recHMWPE (wt%)	Graphene (wt%)
1	35	65	-
2	35	64.5	0.5
3	35	64	1

**Table 5:** Thermal behavior of extruded blends.

Sample	Melt Peak Temperature (°C)	Meling Enthalpy (J/g)	Crystallinity (%)
Reference	129.4	135.3	46.2
0.5% Graphene	131.5	138.5	47.3
1% Graphene	130.6	144.7	49.4

## Data Availability

The original contributions presented in the study are included in the article, further inquiries can be directed to the corresponding author.
